# Single-cell characterization of self-renewing primary trophoblast organoids as modeling of EVT differentiation and interactions with decidual natural killer cells

**DOI:** 10.1186/s12864-023-09690-x

**Published:** 2023-10-18

**Authors:** Bai-Mei Zhuang, Dan-Dan Cao, Tian-Xi Li, Xiao-Feng Liu, Min-Min Lyu, Si-Dong Wang, Xin-Yuan Cui, Li Wang, Xiao-Lin Chen, Xiao-Li Lin, Cheuk-Lun Lee, Philip C.N. Chiu, William S.B. Yeung, Yuan-Qing Yao

**Affiliations:** 1 Shenzhen Key Laboratory of Fertility Regulation, The University of Hong Kong-Shenzhen Hospital, Haiyuan 1st Road, Futian District, Shenzhen, Guangdong P.R. China; 2 Medical school of Chinese People’s Liberation Army, Chinese People’s Liberation Army General Hospital, Beijing, China; 3 Geneplus-Shenzhen Institute, Shenzhen, China; 4 Department of Clinical-Translational and Basic Research Laboratory, The University of Hong Kong-Shenzhen Hospital, Haiyuan 1st Road, Shenzhen, Futian District, Guangdong P.R. China; 5 Department of Obstetrics and Gynecology, Affiliated Shenzhen Maternity & Child Healthcare Hospital, Southern Medical University, Shenzhen, China; 6 Department of Obstetrics and Gynecology, LKS Faculty of Medicine, The University of Hong Kong, Pok Fu Lam, Hong Kong S.A.R.; 7 Department of Obstetrics and Gynecology, The First Medical Centre, Chinese People’s Liberation Army General Hospital, Beijing, China

**Keywords:** Trophoblast organoid, Extravillous trophoblast, Trophoblast differentiation, Placentation, Pregnancy

## Abstract

**Background:**

Extravillous trophoblast cell (EVT) differentiation and its communication with maternal decidua especially the leading immune cell type natural killer (NK) cell are critical events for placentation. However, appropriate in vitro modelling system and regulatory programs of these two events are still lacking. Recent trophoblast organoid (TO) has advanced the molecular and mechanistic research in placentation. Here, we firstly generated the self-renewing TO from human placental villous and differentiated it into EVTs (EVT-TO) for investigating the differentiation events. We then co-cultured EVT-TO with freshly isolated decidual NKs for further study of cell communication. TO modelling of EVT differentiation as well as EVT interaction with dNK might cast new aspect for placentation research.

**Results:**

Single-cell RNA sequencing (scRNA-seq) was applied for comprehensive characterization and molecular exploration of TOs modelling of EVT differentiation and interaction with dNKs. Multiple distinct trophoblast states and dNK subpopulations were identified, representing CTB, STB, EVT, dNK1/2/3 and dNKp. Lineage trajectory and Seurat mapping analysis identified the close resemblance of TO and EVT-TO with the human placenta characteristic. Transcription factors regulatory network analysis revealed the cell-type specific essential TFs for controlling EVT differentiation. CellphoneDB analysis predicted the ligand-receptor complexes in dNK-EVT-TO co-cultures, which relate to cytokines, immunomodulation and angiogenesis. EVT was known to affect the immune properties of dNK. Our study found out that on the other way around, dNKs could exert effects on EVT causing expression changes which are functionally important.

**Conclusion:**

Our study documented a single-cell atlas for TO and its applications on EVT differentiation and communications with dNKs, and thus provide methodology and novel research cues for future study of human placentation.

**Supplementary Information:**

The online version contains supplementary material available at 10.1186/s12864-023-09690-x.

## Background

Placentation is a choreographed process comprised of trophoblast proliferation, differentiation, invasion and uterine spiral artery remodeling [1]. Abnormal placentation could lead to pregnancy disorders including miscarriage, preeclampsia and fetal growth restriction which are life-threatening to the fetus and mother. Thus, understanding the cellular and molecular events during placentation is quite important. The study of human placentation is limited because of ethical concerns. In recent years, different in vitro models have been developed for modeling human placental development [2], among which the three-dimensional (3D) trophoblast organoids (TO) become powerful in their ability to model placental development more physiologically. TO was firstly introduced by Haider et al. using formula containing EGF, A8301 (a TGF-beta inhibitor), Noggin (a BMP inhibitor), CHIR99021 and R-spondin (WNT inhibitors) and prostaglandin E2 [3]. Replacement of Noggin by FGF2 and Y27630 (ROCK inhibitor) was later reported by Turco et al. TOs derived from primary tissue using Turco’s formula can self-renew for a long term, exhibits trophoblast identity with expression of typical identity markers as well as secretion of pregnancy hormones [2], and resembles normal first trimester placentas of human [4]. Moreover, TOs can give rise to different trophoblast cell types such as extravillous trophoblast (EVT) when exposed in appropriate condition [4]. Therefore, TO provides a useful model for investigating human placental development.

EVT, derived from villous cytotrophoblast (CTB) through cytotrophoblast cell column (CCC), has unique human leucocyte antigen (HLA) expression profile (positive for HLA-C, HLA-G, HLA-E and HLA-F and negative for HLA-A and HLA-B), and can finally fuse to produce multinucleated placental bed giant cells (GCs) [5]. EVTs play critical roles during placentation with their decidual invasion, uterine spiral artery remodeling ability as well as immune modulation, dysfunction of which will cause pregnancy complications [6]. Understanding the regulatory program of EVT formation and their communicative mechanism with maternal decidua are critically important. Different types of EVT can be classified based on a location perspective including interstitial EVT (iEVT) and endovascular EVT (eEVT) [6] or implantation-site EVT and chorionic-leave EVT [7]. Both intrinsic program and extrinsic signals from decidual were revealed for EVT differentiation utilizing different in vitro models. For example, NOTCH signaling was found to regulate EVT formation through culture of primary villous CTBs and first-trimester villous explant [8]. Likewise, with TO, removal of WNT activators was found to promote EVTs generation [3]. Despite this, the regulatory program of EVT differentiation remains largely unknown. By modelling EVT differentiation with the previously introduced TO system, together with the single-cell transcriptomic profiling, a comprehensive molecular characterization of EVT differentiation can be accomplished to expand the corresponding knowledge.

Functional EVT primarily interacts with decidual immune cells to exert their immunomodulation function facilitating normal placentation. From the other way around, decidual natural killer cells (dNKs, CD56^bright^CD16^dim^), the leading lymphocytes (70%) at maternal-fetal interface [9], interact with EVT and play a crucial role in uterine spiral artery remodeling during placentation [10]. Decidual NKs are characteristic of expressing perforin, granzymes, the activating receptors NKp30, NKp44, NKp46, as well as NKG2A and NKG2C, but with low cytotoxicity; secreting cytokines to promote trophoblast invasion. Particularly, different HLA molecules on EVT can exert specific immunomodulation functions by binding to specific partners on dNKs [9]. For example, HLA-E on EVT binds to NKG2A/CD94 on dNKs overriding the cytotoxic signals, while HLA-C1 recognizes KIR2DL2/L3/S2 and HLA-C2 interacts with KIR2DL1/S1. The unique HLA-G on EVT acts with ILT2 and KIR2DL4 on dNKs can induce various cytokines secretion for licensing the immune trophism. The regulation of dNK on EVT is evidenced by studies which showed interleukin (IL) -8 and interferon -γ inducible protein (IP) -10 from dNKs stimulated recruitment and migration of EVT through receptors CXCR1 and CXCR3 on invasive EVTs [11]. Although dNK-EVT is critical for placentation and pregnancy, their communicative mechanisms are still not well explored. Moreover, previous mechanistic studies on them are mostly restricted to NK cell lines or 2D co-culture system which is not a physiological state. We speculate that the TO system resembling early placentation could be appropriate for dNK-EVT interaction modelling and mechanistic exploration.

In the present study, TO system was firstly utilized for modelling EVT differentiation and dNK-EVT interaction. We generated the self-renewing TO from human placental villous and differentiated the organoids into EVTs (EVT-TO) under appropriate conditions. The generated EVT-TO was further co-cultured with freshly isolated dNKs (dNKs-EVT-TO). Single-cell RNA sequencing (scRNA-seq) was then applied for comprehensive characterization of TO, EVT-TO and dNKs-EVT-TO co-cultures. Based on the scRNA-seq data, we firstly identified 3 main subtypes of trophoblast: CTB, STB and EVT. Their similarity with in vivo counterparts were supported by Seurat mapping and trajectory analysis. Based on these defined subpopulations, we then substantially explored the transcription factor regulators and regulatory modules in different trophoblasts. Among the uncovered trophoblast differentiation-associated regulons, both known (e.g., ELK1 and FOXM1) and novel regulators (e.g., TEAD2, CLOCK, STAT6 and SMAD3) were revealed. In the dNKs-EVT-TO co-cultures, extensive ligand-receptor interactions (185 significant pairs) were found between EVTs and dNKs subpopulations partially recapitulating in vivo activities. The effects of dNKs on EVTs were also investigated at single-cell level. The results from this study documented a single-cell atlas for the TO system as modelling EVT differentiation and interaction with dNKs, and thus provide new methodology and potential new research targets for future study of human placentation.

## Results

### Establishment of self-renewing trophoblast organoids with EVT differentiation potential

**Fig. 1:**
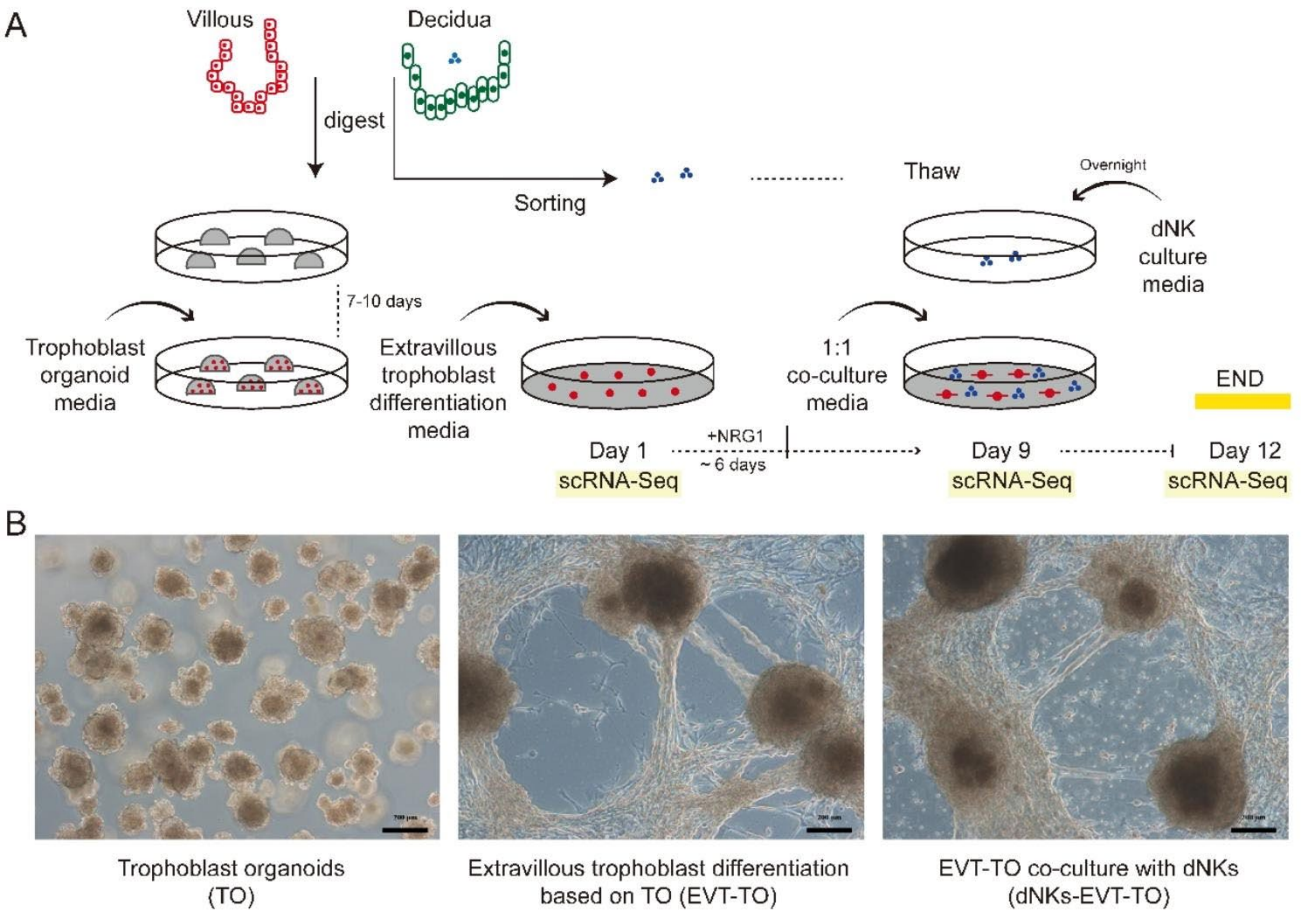
Experiment design of the study. (**A**) Schematic illustration for the formation of the trophoblast organoid, EVT differentiation and co-culture with decidual natural killer cells. (**B**) Image of the different cultured statuses of trophoblast organoids. Left, TO culture; middle, generation of EVTs from TO; right, EVT-TO co-culture with dNKs

**Fig. 2:**
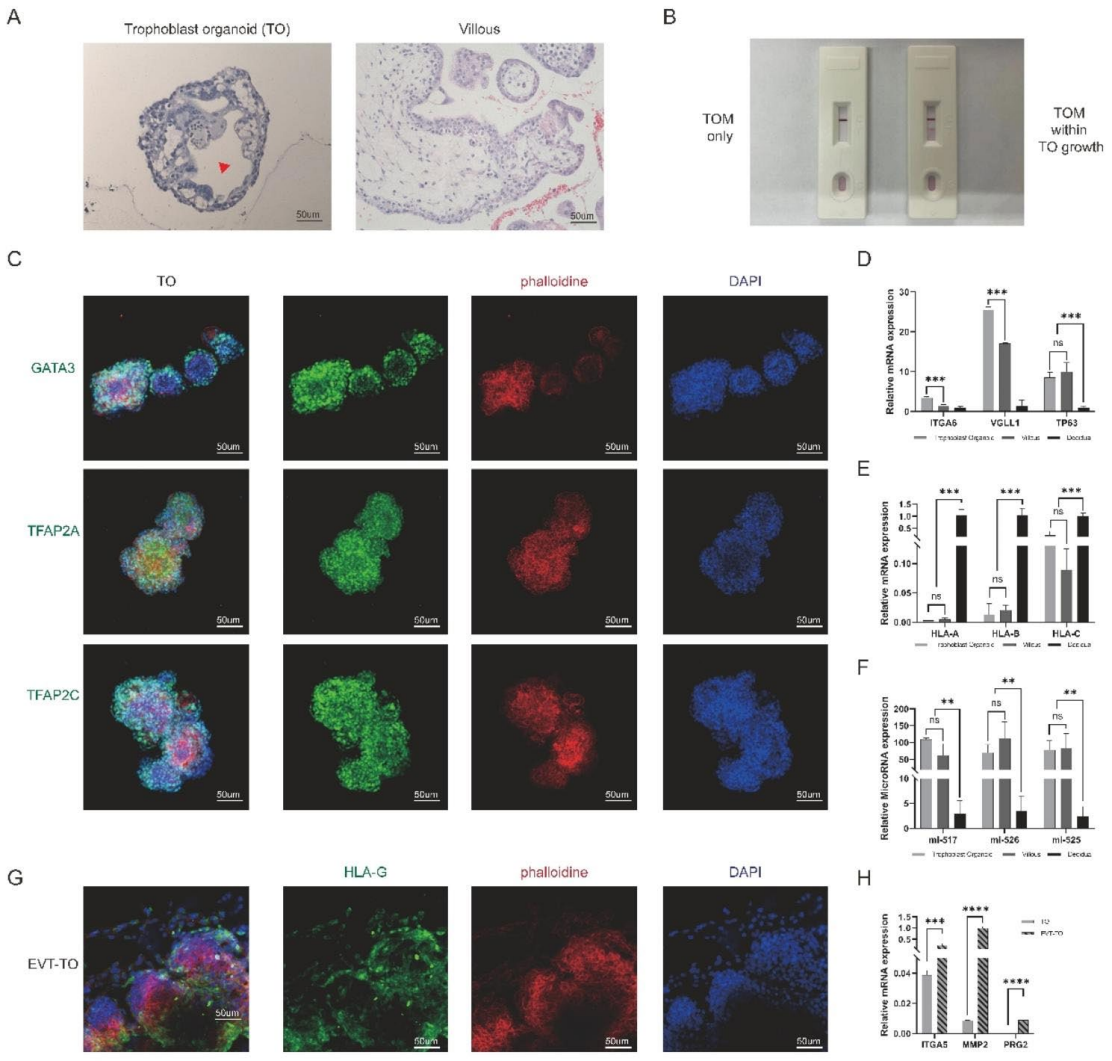
Validation of the trophoblast organoids. (**A**) Images of hematoxylin staining for the cross-section of TO (Left) and first-trimester human placenta (Right). The red arrow indicates the intercellular lacunae. (**B**) Over-the-counter pregnancy test denoting “positive” for the culture medium containing TO growth before next passage. (**C**) Immunofluorescence staining images of GATA3, TFAP2A and TFAP2C in TO. (**D**) Quantification of the relative mRNA expression for ITGA6, VGLL1 and TP63 in TO, compared to first-trimester human placenta and decidua. (**E**) Quantification of the relative mRNA expression for HLA-A, -B, -C and -G in TO, compared to first-trimester human placenta and decidua. (**F**) qRT-PCR analysis on the expression of trophoblast specific microRNAs: miR517-5p, miR-526b-3p and miR525-3p in TO and first-trimester human placenta. Decidua serves as a positive control. (**G**) Immunofluorescence staining images of HLA-G in EVT differentiation stages of TO. (**H**) Quantification of the relative mRNA expression for PRG2, ITGA5 and MMP2 in EVT-TO, compared to TO. **P < 0.01; ***P < 0.001; *ns*, not significant. N = 3 in triplicate

We generated TOs from human placental villous at first-trimester pregnancy according to Turco et al. protocol [12]. In brief, digested tissue was cultured over 7 days before passage in Matrigel-based trophoblast organoid medium (TOM) containing activators (EGF, HGF, FGF2, CHIR99021 and R-spondin-1) and inhibitors (A83-01, Y-27,632) targeting the Wnt, MAPK, cAMP/AKT, TGF-beta, and ROCK signaling pathways (Fig. 1A). The successful establishment of primary human TOs was supported by several lines of evidence (Fig. 1B Left): firstly, the 3D spheroid grows as complex structures with intercellular lacunae (Fig. 2A) and exhibits “pregnant” secretome (Fig. 2B); Secondly, the isolated placental cells form spheroids expressing pan-trophoblast markers GATA3, TFAP2A and TFAP2C (Fig. 2C), and cytotrophoblast progenitor markers ITGA6, VGLL1 and TP63 (Fig. 2D); thirdly, the established TOs were negative for HLA class I molecules (HLA-A, -B and -C) expression (Fig. 2E); fourthly, trophoblast-specific C19MC miRNAs (has-miR-517-5p, has-miR-526b-3p and has-miR-525-3p) were substantially higher in TOs, in accordant with placenta tissue when compared to decidual cells (Fig. 2F). Thus, TO closely recapitulates the organization of placental villous in vivo.

The EVT generation potential from the TOs were subsequently investigated. After several passages, TO culturing medium was changed to EVTM with NRG1 and later without in a sequential fashion (Fig. 1A). By adapting the EVT differentiation protocol, fusiform cells could be observed surrounding the TOs exhibiting migratory history (Fig. 1B Middle). Molecular validation showed that the EVT-specific HLA-G were exclusively expressed in cells growing out from the TOs (Fig. 2G), as well as the up-regulation of other identity genes PRG2, ITGA5 and MMP2 when compared to TO (Fig. 2H). Therefore, TOs could be an effective system as modelling of EVT differentiation.

### Single-cell transcriptomic profiling reveals extensive cellular heterogeneity utilizing the TO system

To identify cellular composition when adopting the TO system, we obtained the transcriptomes of both TO and EVT-TO at single-cell level using 10X Genomic Chromium platform. Standard bioinformatics analysis was performed on the generated single cells (Methods).

**Fig. 3:**
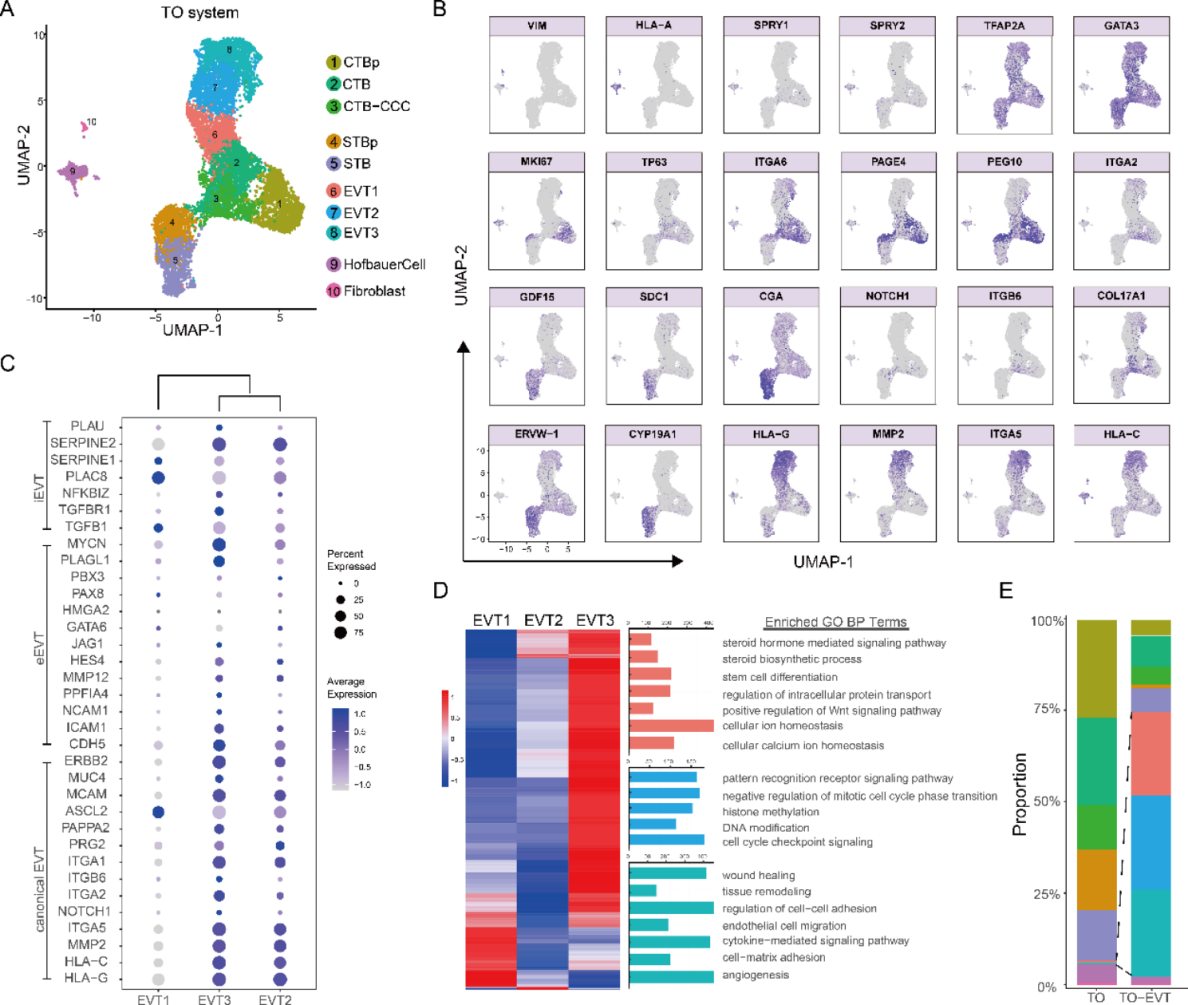
Single-cell characterization of trophoblast organoids (TO). (**A**) Cell clusters for TO sample and EVT-TO sample from 10× Genomics scRNA-seq analysis visualized by UMAP. Colors indicate cell type or state. p, proliferative; CCC, cytotrophoblast cell column. (**B**) Feature plots showing the expression of canonical marker genes for the defined cell types. (**C**) Dot plots showing the expression of known lineage-specific genes for different EVT subtypes. (**D**) GO analysis of the DEGs for the three different subtypes of EVTs. (**E**) Proportion of TO system in each scRNA-seq-defined cluster in the undifferentiation TO state and differentiation EVT-TO state

After quality control, 11,215 cells (5753 cells from TO sample, 5462 cells from EVT-TO sample) were retained for subsequent analysis. As presented in Fig. 3A and B, through manual annotation based on published known markers, 4 major cell types and 10 distinct subpopulations including CTB (CTBp, CTB, CTB-CCC), STB (STBp, STB), EVT (EVT1, EVT2, EVT3) and stromal cells (Hofbauer Cell and Fibroblast) were identified. Specifically, the cell clusters exhibiting high expression of VIM and HLA-A were from stroma cell core. Among these cells, SPRY1 and SPRY2 double-positive clusters were Hofbauer cells, while the remaining termed Fibroblast. Cell clusters with high expression of TFAP2A and GATA3 were cytotrophoblast. These cell clusters were further divided as CTBp with high expression of MKI67 representing the early stage of placental development, CTB positive for ITGA6, TP63, PAGE4 and PEG10 expression and negative for HLA-G expression, as well as CTB-CCC with high expression of ITGA2, COL17A1, NOTCH1 and ITGB6. The cell clusters with high expression of GDF15, SDC1, CGA and ERVW-1 were STB cells. They also expressed mature multinucleated SCT marker, CYP19A1. STB cells were further categorized into proliferative STB (STBp) and STB depending on the expression of proliferative markers MKI67. Cell clusters with high expression of HLA-G, MMP2, ITGA5 and HLA-C were defined as EVT cells, which is further classified into EVT1, EVT2 and EVT3 based on the clustering and UMAP properties.

Detailed analysis showed that EVT1 has lower expression of EVT-canonical markers (e.g., ITGA1, MCAM and ERBB2) and eEVT-related genes (e.g., CDH5, PPF1A4 and JAG1), when compared to EVT2 and EVT3 (Fig. 3C). As to the invasive iEVT-related genes, different EVT clusters has their tendency. EVT3 expressed more of these genes than EVT2. Interestingly, MUC4, which located in implantation site EVT but not in chorionic leave EVT, is highly expressed in EVT3. NCAM1, the typical eEVT gene, was also expressed highly in EVT3. Based on the expression pattern of different EVT-characteristic genes, we inferred that EVT2 and EVT3 might represent the mature EVTs with crucial ability of invasion and artery remodeling. Biological processes for these three EVT clusters were reveled using GO annotation on their differentially expressed genes (DEGs) (Fig. 3D). The GO terms enriched in EVT1 were associated with protein transport, ion homeostasis and stem cell differentiation. EVT2 governed cell cycle checkpoint signaling, while EVT3 manipulated angiogenesis and cell-matrix adhesion. Cell proportion analysis showed that predominant cell clusters of TO contains CTBp, CTB, CTB-CCC, STBp and STB, while in EVT-TO, the major cell clusters changed into EVT1, EVT2 and EVT3 supporting the differentiation from TO (Fig. 3E).

In summary, 10 cell subpopulations were identified as CTBp, CTB, CTB-CCC, STBp, STB, EVT1, EVT2, EVT3, Hofbauer cells and Fibroblast combining the TO and EVT differentiation system. In the TO, an undifferentiated state, CTBs were the major cell types, while upon EVT differentiation, EVTs greatly emerged further supporting the successful system establishment. The presence of CTB, STB and EVT allows us for further investigation of the differentiation regulatory programs.

### Recapitulation of in vivo properties of placentation by the TO system at single-cell level

**Fig. 4:**
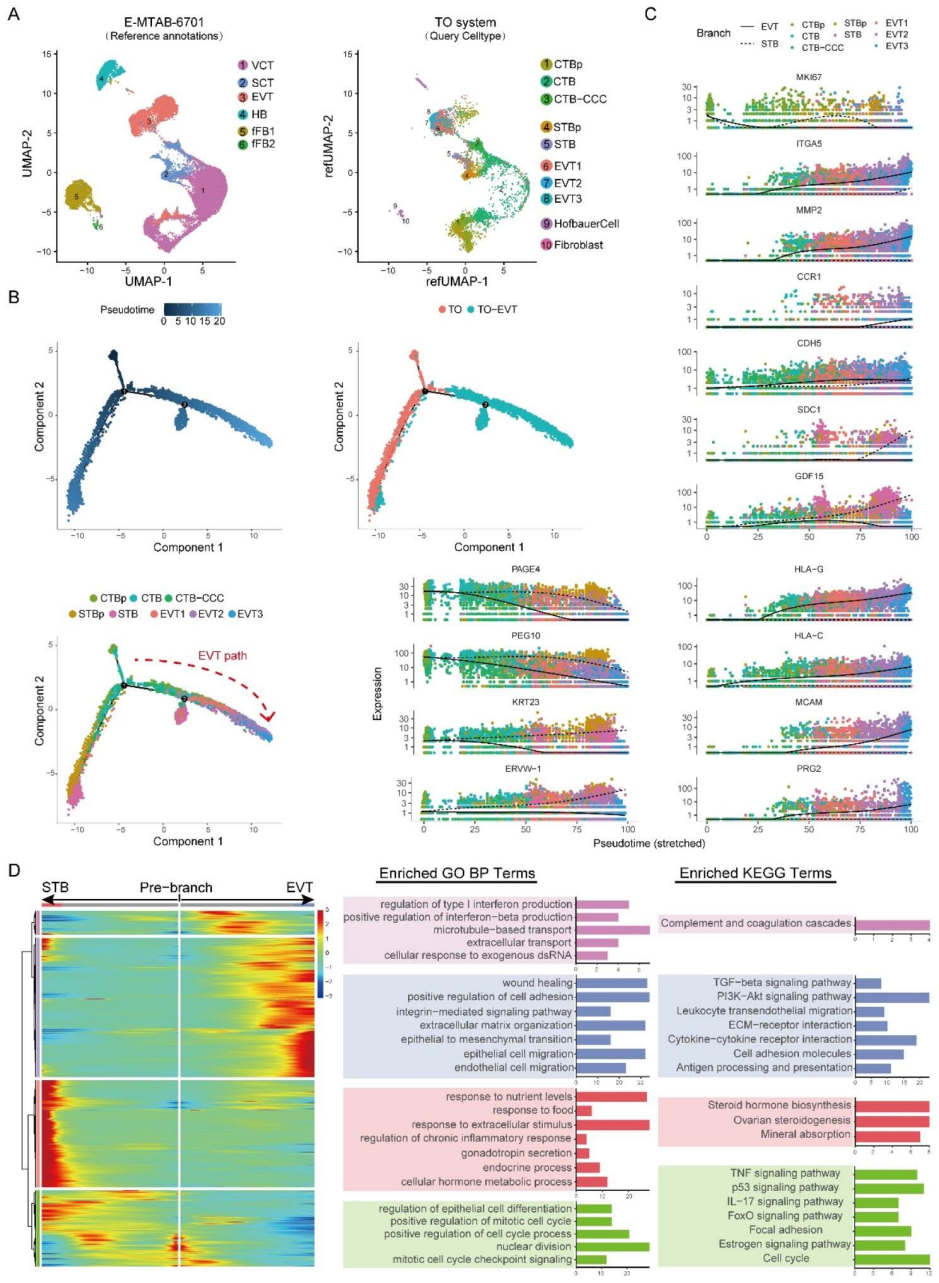
Trophoblasts differentiation trajectories identify a similar pattern of in vivo placentation. (**A**) Annotations query of the trophoblast clusters in the TO system by Seurat mapping analysis. Reference database: the in vivo single-cell dataset of placenta (E-MTAB-6701). (**B**) Pseudotime ordering of the trophoblast clusters indicate the EVT pathways in TO system. (**C**) Pseudotime kinetics of specific representative genes from the root of the trajectory to EVT (solid line) and STB (dashed line). (**D**) Functional enrichment annotations of the clustering branched genes. Left, heatmap showing the genes in trajectory from root to EVT or STB. Middle and Right, Bar plots showing the top annotated GO terms and KEGG terms in four hierarchically clustering genes sets, respectively

We next examined to what extent the TO system recapitulates in vivo properties of placentation by two ways including cell mapping to in vivo single-cell dataset of placenta (E-MTAB-6701) [13] and trajectory analysis. Through Seurat mapping analysis, we found that up to 80% of CTBs, STBs and EVTs in vitro can correctly map to corresponding cell types in vivo, suggesting their resembling characteristics (Fig. 4A). However, CTB-CCC, which highly expressed the markers of CCC in the TO system, was matched more with SCT (also annotation term for STB) in placenta atlas.

Through pseudotime trajectory analysis, a well-ordered differentiation pattern from TOs to EVT-TOs, as well as from CTBs to EVTs and STBs was observed (Fig. 4B). Based on this trajectory, branched expression analysis modeling (BEAM) showed that the proliferative marker MKI67 was upregulated in CTB while downregulated upon differentiation to STB and EVT (Fig. 4C). EVT functional genes ITGA5 and MMP2 for invasion, CCR1 and CDH5 for spiral artery remodeling were all up-regulated in EVT branch; while STB-source hormonal genes SDC1 and GDF15 were up-regulated in STBs branch. Similarly, the other selected traditional trophoblast subtype functional genes were also expressed along the STB or EVT branch in this predicted trajectory. Further functional enrichment annotations of the branching genes additionally verified the reliability of this STB and EVT differentiation trajectory (Fig. 4D). For example, the enrichment of GO terms in EVT branch revealed EVT related activities including extracellular matrix organization, cell adhesion, epithelial to mesenchymal transition and endothelial cell migration; while the GO terms enriched in STB branch were associated with response to nutrient levels, cellular hormone metabolic process and gonadotropin secretion. KEGG analysis enriched well-known EVT-involving signaling pathways “PI3K-Akt” and “TGF-beta” in EVT branch. The ability of the pseudotime trajectory of in vitro TO system to reflect known characteristics/finding of in vivo trophoblast differentiation indicated the utility of TO modeling of human placental development.

Altogether, the TO system, as well as its modeling of EVT differentiation, were characteristic of in vivo trophoblast cells at single-cell transcriptomic level through Seurat mapping and psedotime trajectory analysis. It would be useful for novel regulator discovery during trophoblast differentiation.

### Essential transcriptional regulators for different trophoblast cells

Transcription factors (TFs) regulate gene programs and thereby are targets for controlling cell differentiation. Single-cell TF regulatory networks were analyzed by SCENIC in the TO system to explore the possible TFs, which participated in cell identity maintenance with major focus on TFs regulating EVT differentiation.

**Fig. 5:**
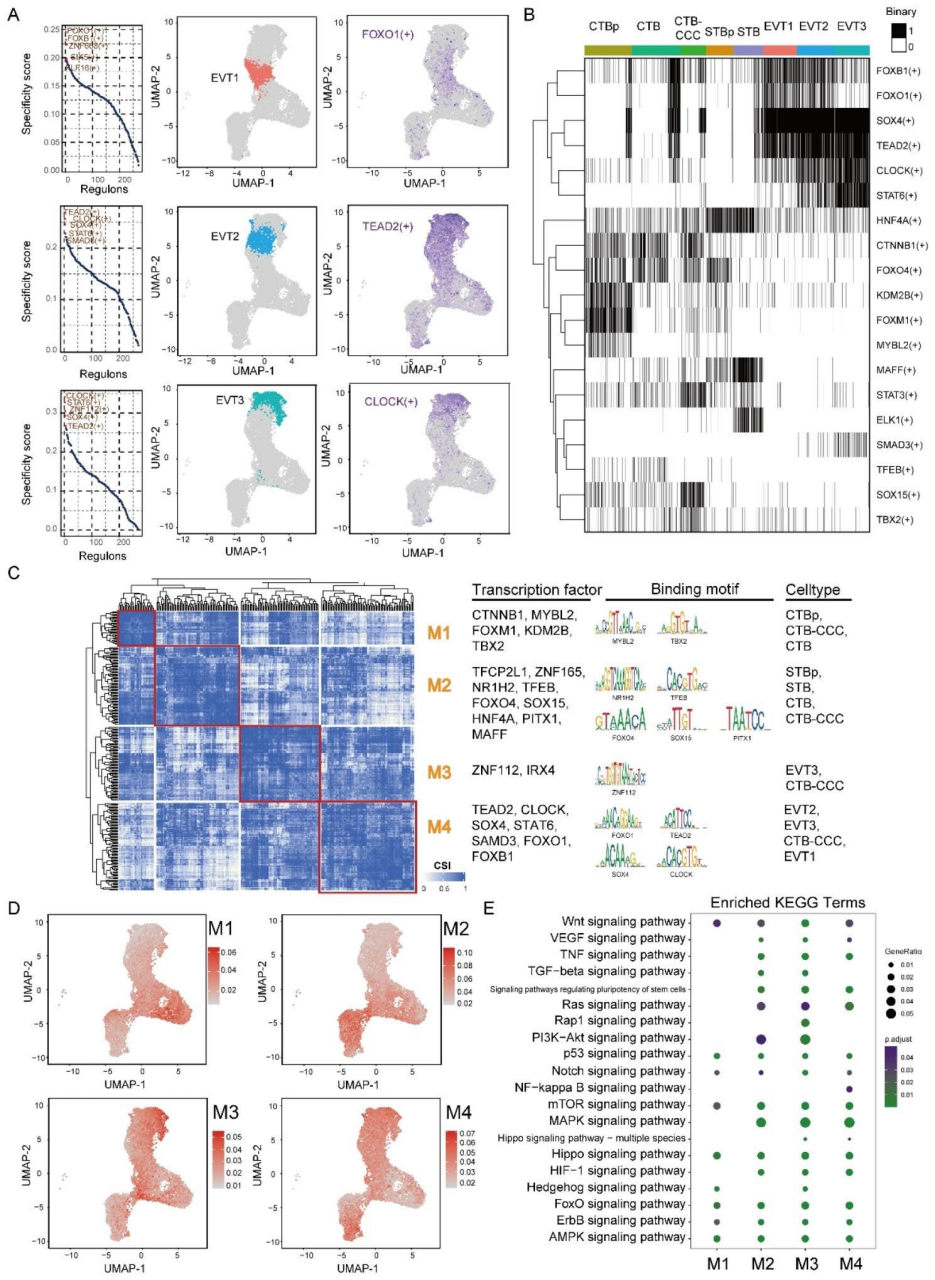
Transcriptional regulators analysis for TO system. (**A**) EVTs-specific regulon activity analysis. Left, Rank for regulons based on regulon specificity score (RSS); Middle, EVT clusters are higjlighted in the t-SNE map (colors dots); Right, Binarized regulon activity score (RAS) for regulons in the t-SNE map (purple dots). (**B**) Binary activity matrix for cell types-specific regulons. Regulons were determined to be active (black) if they exceeded a manually adjusted AUC regulon-specific threshold, or inactive under this threshold (white). (**C**) Identified regulon modules based on regulon connection specificity index (CSI) matrix, along with representative transcription factors, corresponding binding motifs, and associated cell types. (**D**) Average module activity scores mapped on t-SNE. (E) KEGG terms for the enrichment of downstream targets of each module regulators

In total, 270 regulons were identified among the above 8 trophoblast clusters. Regulon activity score (RAS)-based clustering of the cells identified three main trophoblast types: CTB, STB, and EVT. To obtain the cell clusters-specific TFs, we then count the regulon specificity score (RSS) for each trophoblast clusters (Fig. 5A, S1A and S1B). The cluster-specific regulon activity was also demonstrated by tSNE plot. Among the identified top regulons for each cell type/cluster, we successfully identified known regulators with literature evidence. Of note, ELK1, the STB-specific regulon predicted here, was reported to induce BeWo cell (representative STB-like cell line) differentiation via galectin-1 and MAPK pathway [14]. FOXM1, another published gene involved in trophoblast proliferation and migration, ranked the top in CTBp-specific regulons [15]. Interestingly, our data also revealed strong novel regulon candidates that may regulate trophoblast cell differentiation/function. For example, top-ranking regulons in EVT clusters such as FOXO1, FOXB1, TEAD2, CLOCK, SOX4, STAT6 and SMAD3, could be essential EVT differentiation controllers. To further verify the accuracy of the predicted cluster-specific regulons, we also evaluated the openness of regulons and found they were indeed activated in their subordinative trophoblast subpopulations (Fig. 5B). For example, comparable activity of the three EVT clusters was SOX4, TEAD2, STAT6 and CLOCK. Moreover, we also count the RSS for each group in the TO system and found that the TO specific regulons contain TFCP2L1, TFEB, PITX1, FOXI3 and MSX2, while the EVT-TO specific regulons include SOX4, TEAD2, STAT6, SMAD3 and IRF3. Thus, the predicted EVT-specific regulons were stable disturbed both in cell types or groups (Figure S1C and S1D).

To reveal TFs that work in combination in the trophoblast cells, we compared the RAS similarity of each regulon pair based on the Connection Specificity Index (CSI) described as in a previous study [16], and identified 4 major modules among the 270 regulons (Fig. 5C and S2A). Representative regulators and associated cell types were identified in each module through average activity scores (Fig. 5D and S2B). Specifically, M1 module which is associated with CTBp contains regulators TEAD4, a TSC marker, FOXM1, regulator for trophoblast proliferation and CTNNB1, the key effector molecules of WNT signaling pathway. M2 module contains regulators CDX2, MSX2, ETS2, TFAP2C related to TSC self-renewal and stemness, STAT3, NFKB2 related to embryonic stem cell self-renewal and stemness, and FOSL1 related to CTB proliferation. KEGG enrichment analysis of downstream targets of M2 module regulators showed their involvement in signaling pathways targeting by the TOM components (e.g., MAPK, TGF-beta, Wnt and PI3K-Akt) (Fig. 5E). This module was principally associated with CTB- and STB-related clusters. M3 module exhibits higher specificity for EVT1, EVT2, EVT3 and CTB-CCC but contains few known regulators for EVT function/differentiation, expect MYC. Differently, M4 module significantly associates with EVT related clusters and contains well-known EVT regulators including SOX4, GCM1, ETS1 and SMAD3. SOX4 shapes the BMP2-regulated transcriptional network during invasive trophoblast differentiation [17]. GCM1 facilitates the transition from a proliferative to a differentiation phenotype in both villous CTB and EVT [18]. SMAD3 regulates EVT differentiation through TGF beta signaling pathway [19]. M4 module also contains GATA2, TFAP2A and DLX3 which are associated with STBs. Downstream targets of M4 module regulators enriched in top-ranked signaling pathways including “FoxO”, “MAPK” and “HIF-1”. “FoxO” [20] and “MAPK” [21, 22] signaling pathways were reported to regulate trophoblast migration and invasion; while HIF-1 signaling pathway was crucial for trophoblast differentiation in rodents [23]. Additional enriched pathways in M4 module includes “ErbB”, “Notch”, “Wnt”, “VEGF” and “NF-kappa B” signaling pathways, implicated in the regulation of EVT differentiation (Fig. 5E). Therefore, regulators in M4 module and their significantly enriched signaling pathways deserve further functional study for their role in maintaining EVT identity.

By analysis of trophoblast subtype-specific TFs activation, the stability of EVT differentiation in the TO system was further clarified. TO with the ability of EVTs generation would be an emerging approach for placental study.

### Application of TO system to study decidual NK-EVT interaction

Decidual NKs are the predominant immune cells at maternal-fetal interface. Their communication with EVTs promotes cytokine production, trophoblast invasion and spiral artery remodeling facilitating pregnancy. Previous models studying dNK-EVT interaction mostly used cell lines or in a 2D way and seemed to fail to holistically reflect in vivo properties. To complement these drawbacks, we tried a co-culture model utilizing freshly isolated dNKs and EVT-TOs (Methods, Figure S3 and 1B Right). Single-cell transcriptomic investigation of this co-culture model was further performed.

#### Identification of subpopulations in dNKs-EVT-TO co-cultures

**Fig. 6:**
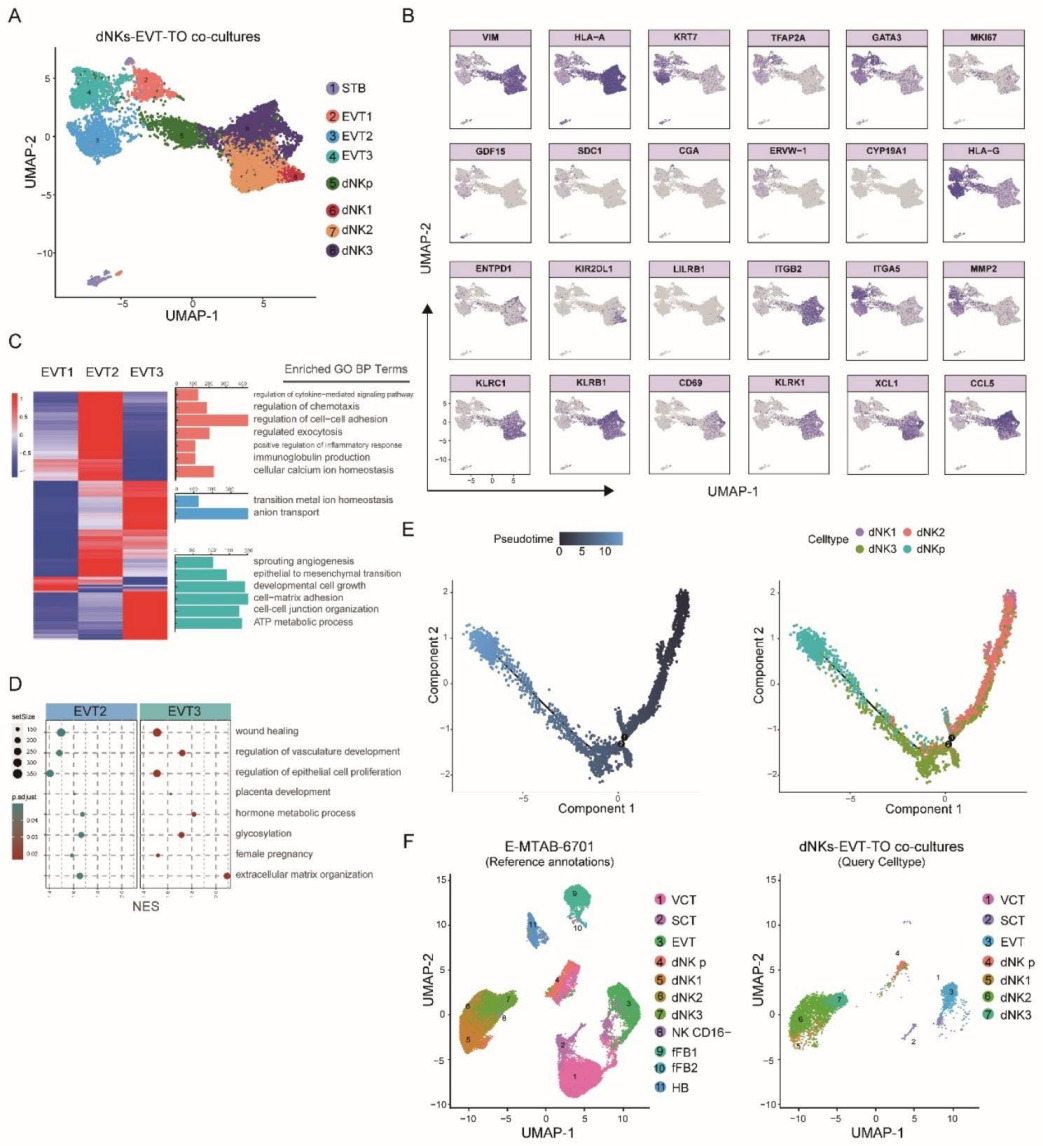
Single-cell characterization of dNKs-EVT-TO co-cultures. (**A**) Cell clusters for dNKs-EVT-TO sample from 10× Genomics scRNA-seq analysis visualized by UMAP. Colors indicate cell type or state. (**B**) Feature plots showing the expression of canonical marker genes for the defined cell types. (**C**) GO analysis enriched different terms on the DEGs for the three different subtypes of EVTs. (**D**) GO analysis enriched shared terms in EVT2 and EVT3. (**E**) Pseudotime ordering of the trophoblast clusters indicate the dNKs pathways in dNKs-EVT-TO co-cultures. (**F**) Annotations query of the trophoblast clusters in the dNKs-EVT-TO co-cultures by Seurat mapping analysis. Reference database: the in vivo single-cell dataset of placenta (E-MTAB-6701)

Same analysis procedure as previously described was applied. In total, 8 distinct cell populations were identified with 8283 high-quality cells (2932 cells from EVT-TO sample, 5165 cells from dNKs sample) (Fig. 6A). Among these 8 populations, 4 non-trophoblast cell populations, names proliferate dNK (dNKp) population and three dNK clusters (dNK1, dNK2, dNK3), were revealed with high expression of VIM and HLA, and, 4 trophoblast subpopulations (STB, EVT1, EVT2 and EVT3) were annotated with expression of KRT7, TFAP2A and GATA3 (Fig. 6B). STB cluster expressed GDF15, SDC1, CGA, ERVW-1 and CYP19A1. EVT subpopulations expressed HLA-G, ITGA5 and MMP2. According to the EVTs annotations in the TO system described previously, here, the EVT subpopulations were also divided into three clusters. GO enriched analysis was then performed for DEGs of these three EVTs clusters and reveled that they governed different biological processes (Fig. 6C): EVT1 was associated with regulation of chemotaxis, exocytosis and inflammatory response, whereas EVT2 controlled anion transport and metal ion homeostasis; EVT3 manipulated sprouting angiogenesis and epithelial to mesenchymal transition. Furthermore, placenta development and female pregnancy were enriched in EVT2 and EVT3, suggesting these two EVTs subpopulations contributed mostly in conventional trophoblast function (Fig. 6D).

Four main dNK subpopulations were defined according to two single-cell transcriptome studies about human first-trimester placenta and decidua tissue [13, 24]. dNK1 expressed ENTPD1, KIR2DL1 and LILRB1, dNK2 and dNK3 both highly expressed ITGB2, but only dNK2 highly expressed KLRC1 and only dNK3 highly expressed KLRB1, CD69 and KLRK1 (Fig. 6B). In addition, the expression of known NK cell-derived chemokines was also used for dNK subsets identification. Both dNK2 and dNK3 were expressed high levels of XCL1, and CCL5 is highly expressed by dNK3. Moreover, pseudotime trajectory analysis for dNK clusters reveals a well-ordered differentiation pattern from dNK1 to dNK2 and then to dNK3 and dNKp (Fig. 6E).

We also explore the similarity between the single cells of dNKs-EVT-TO co-cultures and the corresponding cell types in vivo at transcriptomic level, and found that up to 77% of these cell clusters can correctly mapped (Fig. 6F). These evidences altogether demonstrated that this in vitro dNKs-EVT-TO co-cultures could be utilized to study dNK-EVT interactions.

#### Cell-cell interactions between EVTs and dNKs

**Fig. 7:**
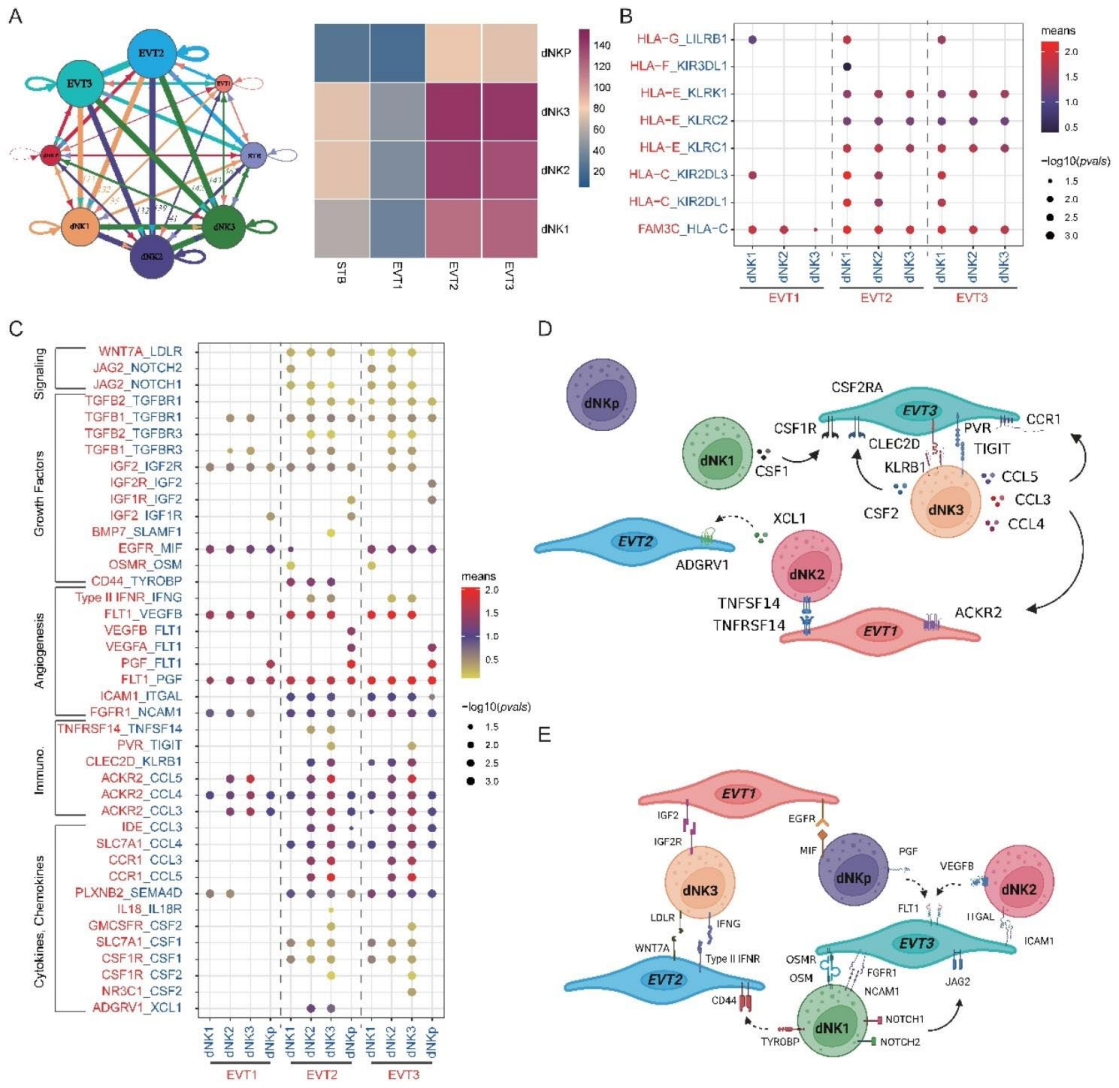
Cell-cell interactions in dNKs-EVT-TO co-cultures. (**A**) Putative ligand and receptor-based intercellular communication between dNKs and EVT-TO cell types. Left, circus plot. Color lines indicate ligands broadcast by the cell population of the same color and connect to cell populations where cognate receptors are expressed. The line thickness is proportional to the number of ligands where cognate receptors are present in the recipient cell population. Loops indicate autocrine circuits. Map quantifies potential communication, but does not account for anatomic position or boundaries of cell populations. Right, Heatmap for counts of predicted pairs. (**B**) Overview of HLA molecules-representative ligand-receptor interactions. *P-*values < 0.05 indicated by circle size. The means of the average expression level of cell-cell pairs indicated by colors. (**C**) Overview of selected ligand-receptor interactions. Immuno., immune checkpoints. (**D**) Diagram of the main receptors and ligands expressed on dNKs subsets and EVTs clusters that are involved in cytokines, chemokines and immune checkpoints. (**E**) Same as D but for angiogenesis, growth factors and signaling

Utilizing the single-cell data, a systematic exploration of interactions between EVTs and dNK subpopulations was further performed through CellPhoneDB analysis. The circos plot detected broadcast ligands and demonstrated extensive communication for cognate receptors (Fig. 7A Left). Notably, dNK3 showed the most of interactions with EVT2 and EVT3, followed by dNK2 and dNK1. The interactions of dNKp with EVTs were weak, but relatively stronger dNKp and EVT2/3 were also observed (Fig. 7A Right).

We firstly checked the HLA molecules in the resulted ligand-receptor pairs. Among the HLA molecules, EVT-source HLA-G only interacted with dNK1-source LILRB1 at a significant level. Other HLA molecules in EVTs including HLA-F, HLA-E and HLA-C was found to interact with KIR3DL1, KLRK1 and KLRC1/2, KIR2DL3 and KIR2DL1 in dNKs, respectively at statistically significant level (Fig. 7B). Interestingly, dNKs can provide HLA-C to bind the FAM3C expressed in EVTs.

Then, 185 significant stronger cell-cell interactions between EVTs and dNKs were predicted. These ligand-receptor complexes were mostly concentrated between dNK1/2/3 and EVT2/3, relate to cytokines, chemokines, immunomodulation, angiogenesis, growth factors (Fig. 7C, D and E). Some of the interaction patterns were consistent with the in vivo properties [13]. For example, CSF1 was highest expressed in dNK1, interacting with its receptors CSF1R expressed in EVT2 and EVT3 (Fig. 7D and S4). XCL1 was expressed higher in dNK2/3 but only significantly interacted with ADGRV1 expressed on EVT2, other than its canonical receptor XCR1. The ligand-receptor complex CCL5-CCR1 and CCL3-CCR1 were predicted between dNK2/3 and EVT2/3, suggests a role for dNK2/3 in regulating EVT2/3 invasion. Surprisingly, MIF, rather than AREG, was predicted to communicate with EGFR expressed in EVT1/3, potentially suggesting that dNKs might also negatively regulate EVTs development in addition to their positive support for EVT [25]. Putative inhibitory interactions in dNK-EVT were also identified, including dNK3-expressing KLRB1 and TIGIT coupled with EVT3-expressing CLEC2D and PVR. Furthermore, previously predicted maternal-fetal interface interacting pairs ACKR2-CCL3/4/5, OSMR-OSM, FLT1-VEGFB and FGFR1-NCAM1 were also identified [13] (Fig. 7E and S4). The cytokine involved interactions CCL3-IDE and CCL4-SLC7A1, functioning in hematopoietic stem cell and multipotent progenitor cell niche, were also activated in our dNKs-EVT-TO co-cultures [26]. Unreported dNK-EVT interacting molecules were additionally revealed by this study, such as TGFBR1-TGFB1 and IGF2-IGF2R/1R, LDLR-WNT7A and NOTCH1/2-JAG2, which were involved in cell growth and EVTs differentiation, respectively.

Overall, EVT2 and EVT3 showed stronger interactions with dNK2 and dNK3 mostly related to cytokines, immunomodulation and angiogenesis, while EVT1 mainly participated in regulation of angiogenesis. With our dNKs-EVT-TO co-culture model, extensive dNK-EVT interacting pairs were identified which might help uncover the modulating mechanisms of dNKs and EVTs during placentation. Further functional study of those pairs at maternal-fetal communication should be warranted.

#### Effects on EVTs exerted by dNKs in the co-cultures

The above prediction of strongest interactions in EVT2/3-dNK1/2/3 conversation and EVT2/3-dNKp partnership led us to wonder whether there were effects caused by dNKs to these EVT subpopulations. Through comparison analysis to the EVT clusters in the absence of dNKs, we found significant DEGs for EVTs in the dNKs-EVT-TO co-cultures.

**Fig. 8:**
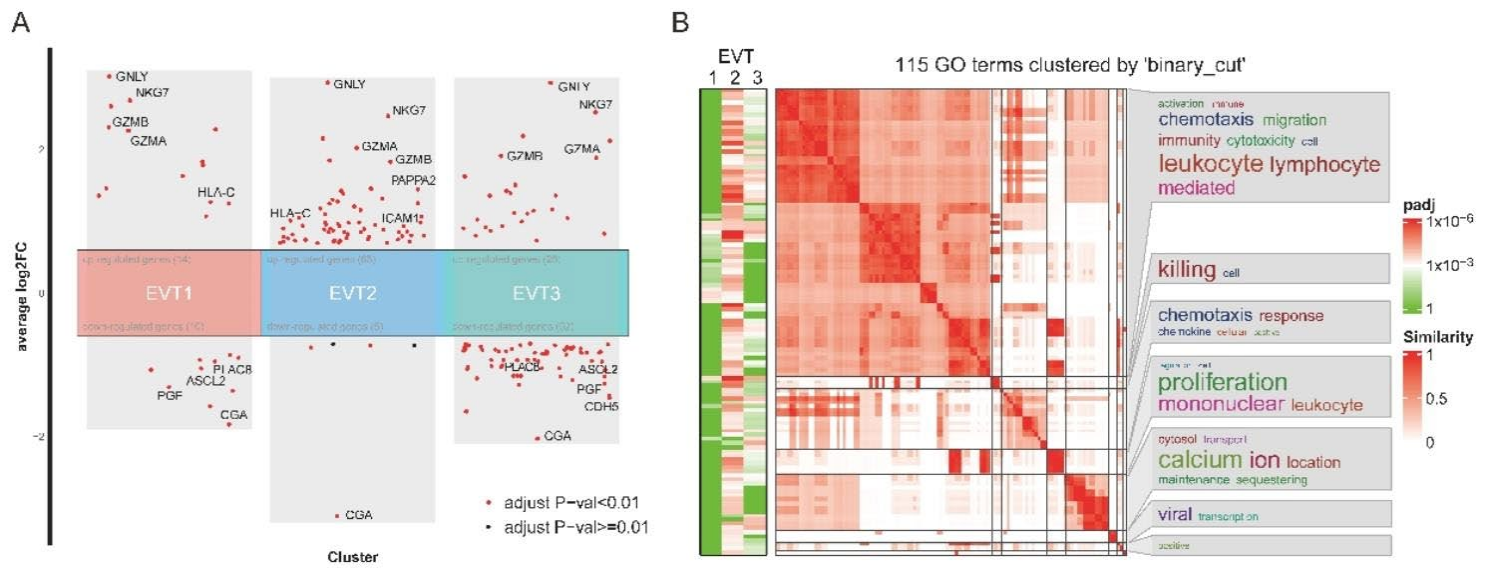
Effects on EVTs exerted by dNKs in the co-culture system. (**A**) Volcano plot showing DEGs for the three different subtypes of EVTs. (**B**) Simplified GO terms enrichments for DEGs as A

Compared to EVT1, dNKs caused much more expression changes in EVT2 and EVT3 given that they had higher number of DEGs (Fig. 8A). Interestingly, the cytoplasmic granule genes (GNLY, NKG7, GZMA and GZMB) were all up-regulated in each EVT subpopulations when co-cultured with dNKs, suggesting dNKs caused a universal effect to the EVTs.; whereas the hormone genes CGA and PGF were all down-regulated in each EVT subpopulations. In addition, expression of angiogenesis inductor (ICAM-1, CDH5) and epithelial-mesenchymal transition (EMT) related genes (PLAC8 and ASCL2), which involves in regulating EVT function were also changed in the presence of dNKs. HLA-C, one of the EVT-specific major histocompatibility complex (MHC) molecules extensively studied in pregnant reject disorders [27], was found to be upregulated in EVT1 and EVT2, indicating immune response induced by dNKs. Biological processes significantly enriched out by GO analysis were mostly in EVT2, converging on chemotaxis, transport for cytosol and calcium ion, and proliferation for mononuclear and leukocyte (Fig. 8B).

In this section, the results showed that transcriptomic changes in EVTs were caused by dNKs in our in vitro co-cultures, even with a relative short duration (co-culture for 3 days) indicating the effectiveness of this dNK-TO-EVT co-culture system. However, what will happen in a longer co-culture way and the mechanism how dNKs affect EVT function should be further explored.

## Discussion

Trophoblast organoid is one of the in vitro culture systems advancing the placentation research [7]. This organoid system was considered superior to previous conventional human trophoblast lines (e.g., immortalized trophoblast cell lines, pluripotent stem cell-derived trophoblast) and placental villous explants. The present study firstly established the TO and EVT generation according to the protocols from the Nature paper, and with the scRNA-seq, comprehensively characterized the TO and TO-EVT which showed their extensive resembling to the in vivo properties of first-trimester pregnancy-derived placental villous. With the TO and TO-EVT system, transcription factors for trophoblast differentiation were then investigated at single-cell level. Both known and novel regulators for maintaining trophoblast subtypes-associated feature were revealed. Finally, an EVT-TO-dNK co-culture system was firstly tried and examined their possibility to recapitulate the in vivo dNK-EVT crosstalk. As expected, the ligand-receptor interactions between EVTs and dNK1/2/3 in our dNKs-EVT-TO co-cultures can basically mimic the in vivo dNK-EVT communication, for example, the HLA profile-associated interactions. Hence, TO will be a promising system for benefiting the future mechanistic study of maternal-fetal interface and disorders.

In vitro culture of trophoblasts is a key obstacle for placentation study, especially for primary EVTs. Isolated from the first-trimester placental villous, primary trophoblast cells are vulnerable and poorly adhere to plastic after 24 h. After 3–4 days, they cease to proliferate, lose natural phenotype and overgrow with mesenchymal cell contaminants. Until 2018, EVT differentiation medium containing NRG1 and A-8301 together with Matrigel, was successfully developed based on primary CT-derived trophoblast stem (TS) cells and blastocyst-derived TS cells [28]. This EVT medium was also used in generating EVTs from TO, the self-renewing 3D spheroid with fully characterization of the trophoblast criteria [4]. With the medium, the long-term cultured TO can generate EVT after 3-7days in vitro characteristic of HLA-G^+^ expression and vigorously invading and digesting Matrigel in 3D. Nevertheless, application of TO were confined to morphogenetic events for CTB and STB [2, 29] instead of EVT differentiation. The present study fully utilized the TO system in the modelling of EVT differentiation as well as EVT interaction with dNKs. With the TO and TO-EVT, at single-cell level, we identified three unique EVT states, which differentiated from our 3D organoids, that were distinguishable by state of metabolism (EVT1), cell cycle checkpoint signaling (EVT2) and angiogenesis (EVT3). Concurrent pseudotime trajectory analysis identified the EVT differentiation path from EVT1 through EVT2 to EVT3. These EVT clusters might represent snapshots of transient cell states or the spatial distance captured but no specific cell types per se during placentation. The represented genes expression in our EVT clusters indicates the presence of eEVTs which was missed by a recent published article that only found iEVTs [30]. It might be caused by that our EVTM2 is without Matrigel. Endovascular EVTs initially form arterial plugs and eventually replace the maternal endothelium. Since the expression of extracellular matrix (ECM) component was visualized in the arterial plugs in the in vivo spatial transcriptomics, we infer that eEVT development should firstly conquer to go through the hindrance of ECM component. Thus, the concentrations of Matrigel, which as the fungible ECM, should be paid more attentions for EVT in vitro culture.

Transcription factors coordinated diverse signaling cues to collectively determine the cell type-specific characteristics [31]. Our study expands the description of TFs associated with trophoblast differentiation in an in vitro culture. We focused mostly in TFs associated with EVT differentiation and found some novel regulons (e.g., FOXO1, TEAD2, CLOCK and SOX4). Take FOXO1 for an example. Low expression of FOXO1 was evaluated in preeclampsia. Up-regulated FOXO1 would enhance migration and invasion, and inhibit apoptosis of trophoblast through activating AKT signaling pathway thus for alleviating preeclampsia [32]. Down-regulated FOXO1, which effected by oxidative stress, could impaired trophoblast adhesion and migration via mediating expression of integrin β3 in preeclampsia [33]. Therefore, whether FOXO1 is relate to EVT differentiation deserves further exploration, so does other discovered regulons. TFs modules analysis indicated an elaborate sequence of trophoblastic genes that work synergistically in fine-tuning cellular states. The signaling pathways enriched out from downstream targets of these TFs modules are involved in different trophoblast subtypes. M3 and M4 module were relatively specific to EVT. Hippo signaling pathway is specifically shared by these two modules. It has been studied to organize EVT lineage fate currently [34]. That might further reinforce the reliability of TFs module related to trophoblast subtypes. “Ras” signaling pathway were unique to M3 module, as well as “NF-kappa B” to M4 module. “Hedgehog” was mutual to M1 and M3 module, while “TGF-beta” was owned by M2 and M3 module (Fig. 5E). TGF-beta superfamily members exert a huge character in regulating EVT invasion, though, with conflict results [35]. Although debatable, the suppressive function of TGF-beta for EVT invasion was supported by the majority of reports. A83-01, a TGF-beta inhibitor, is, indeed, used in inducing EVT generation [12, 36, 37]. The correlation of the unique or mutual signaling pathways for these TFs modules, as well as their affiliated TFs, would provide effective information for trophoblast identifies and contribute to improve the media component for maintaining the EVT natural phenotype as possible during the in vitro long-term culture.

Diverse partnership derived from maternal endometrium is another influence for EVT development. Decidual natural killer cells are the chief nanny at maternal-fetal interface during placentation. Its involvement in spiral arteries remodeling under coordination with EVTs are always heated studied. Our study tried to repeat this dNK-EVT crosstalk using an in vitro co-culture model utilizing the organoid system. Trophoblast has a unique HLA profile: HLA-C, -G, -E and -F were up-regulated only with differentiation to EVT, HLA-A and -B remained permanently silenced. Most of the communications between HLA molecules and their receptors can be recapitulated in our dNKs-EVT-TO co-cultures, including the famous HLA-G-LILRB1. However, we also found interesting new interactions such as FAM3C and HLA-C. FAM3C is a cytokine triggering the EMT program and regulate various proteins including Ras, STAT3, TGF-β and LIFR. Down-regulated FAM3C reduced the invasive outgrowth of lung tumor cell lines by inhibiting proliferation, MMP expression and E-cadherin membrane localization [38]. Uptake of FAM3C through circulatory extracellular vesicles was able to interact with Ras-related protein RalA to activate the downstream Src/Stat3 signaling cascade for participating the EMT process [39]. The EVT differentiation, somewhat, seems to undergo EMT. Therefore, the relevance of FAM3C with placentation could be high. HLA-C is another MHC molecule highly expressed in EVT, but here also detected in dNKs to significantly interact with EVT-derived FAM3C. dNKs, itself, has two KIR-binding epitopes of HLA-C: KIR2DL1 and KIR2DL3 [40]. This recognition of self-MHC class I (MHC-I) via inhibitory NK cell receptors is termed as NK cell education, which prevents NK cell auto-reactivity and maintains tolerance to itself [41]. But NK education does not exclusively rely on KIRs. It also can be calibrated by NKG2A/HLA-E engagement [42]. Moreover, dNKs can acquire surface HLA-G by antigen presentation and trogocytosis [43]. Whether HLA-C could act in a similar path to HLA-G cycle, or how it complicatedly correlates with FAM3C via KIRs, were an interesting topic. The other known ligand-receptor interactions for EVTs-dNKs are also predicted in this in vitro co-culture study. Our cellphoneDB findings suggest a similar in vivo environment coordinating multiple communicating modulatory pathways. We also tried to seek expression changes in EVTs by the influence of dNKs and found that dNKs really exerted, albeit seemingly slight, effects to EVT development.

## Conclusions

In summary, we generated EVTs from TO and then built a dNKs-EVT-TO co-culture system for the identification of molecular and cellular mechanisms that operate an in vitro trophoblasts environment. ScRNA-seq was used in our study to define EVT differentiation trajectories and the TFs coordination in our in vitro human placentation model. The dNK-EVT communication was chosen to reproduce known interactions and predict new interactions under a co-culture condition. Importantly, we described comprehensively the self-renewing TO, derived from first-trimester pregnant villous, as a well-designed modeling of EVT differentiation and interactions with dNKs. This cell atlas provides valuable resources for further application on placentation mechanism researches. However, the human tissue used for TO construction and subsequent scRNA-seq in our study was only originated from one healthy pregnant woman. Future increase of the sample source is required and follow-up functional experimental exploration would warrant.

## Materials & methods

### Tissue collection

This study was approved by the Ethics Committee of the University of Hong Kong-Shenzhen Hospital (IRB no. [2021]172). Informed consent was obtained from all the participants enrolled in the study.

Human placenta and decidual tissues were obtained from normal first-trimester pregnant women who voluntarily underwent elective termination of pregnancy. All the tissues were disposed according to the protocol of Male V et al. [44].

### Trophoblast organoid formation, culture and EVT differentiation

Placenta villus was washed in PBS for at least 10 min to remove maternal blood contamination before processing. After scraped from the chorionic membrane, the tissue was enzymatically digested in 0.2% trypsin-250 / 0.02% EDTA / PBS solution at 37 ℃ for 10 min. The cell suspension was filtered through 100-µm cell sieve, centrifuged at 600 g for 6 min and then mixed with ice-cold Matrigel at a ratio of 10× volume: 1× volume. The Matrigel / cell suspension was pipetted into 48-well plate with 25 µl drop each well for routine culture. Primary trophoblast organoids (primary TO) formed in 7–10 days. Trophoblast organoid media (TOM) were refreshed every 3–4 days. At passage 3, primary TO were subjected to EVT generation medium (EVTM) for the test of differentiation potency.

The above culture media formula was from that of Turco MY et al. and Okae et al. [12, 28]. EVTM is a sequential treatment with EVT basic medium plus NRG1 (100ng/ml) and Matrigel (0.5%) for the first 6 days followed by EVT basic medium only for the further 3 days.

### Decidual natural killer cells (dNKs) isolation and cryopreservation

Decidual tissues were cut into approximately 0.2-mm^3^ pieces and then enzymatically digested in 1 mg/ml collagenase V solution at 37 ℃ for 60–70 min. The supernatant was passed through orderly 100-µm and 40-µm cell sieve, and then resuspended in red blood lysis buffer for 10 min. After washing and centrifugation, the leukomonocytes were isolated and incubated in a T75 flask with RPMI 1640 medium for 2 h at 37℃. The non-adherent cells were collected for fluorescence-activated cell sorting with a BD FACSAria II Flow Cytometer (488/633/405), which were incubated with the 7-AAD staining solution (1:400, BioLegend, USA), Brilliant Violet 421 Annexin V (1:200 BioLegend, USA), Alexa Fluor® 700-conjugated anti-human CD45 (1:200 BioLegend, US), APC/Cy7-conjugated anti-human CD56 (1:200 BioLegend, USA), PE-conjugated anti-human CD16 (1:200 BioLegend, USA) and FITC-conjugated anti-human CD9 (1:200 BioLegend, USA) for FACS. Primary dNKs with > 95% purity was obtained for further experiments.

After culture in dNKs medium which contained X-Vivo supplementation (Lonza, USA) of 5% human AB serum and 40 ng/ml IL-15 overnight, the primary dNK cells were divided into two parts and cryopreserved for the further co-culturing or flow cytometric analysis.

### Co-culture of EVT-TO and dNKs

Before co-culture, four wells of primary TO were passaged and transferred into a 35 μm dish pre-coated with 1% Matrigel for the EVT differentiation with the sequential EVTM treatment for 9 days. The cryopreserved dNKs were thawed and culture overnight before co-culture. Then, 2 × 10^5^ dNKs in 1 ml EVTM (without NRG1) and 1 ml X-Vivo plus supplements were added to the 35 μm dish. After 72 h of incubation, the supernatant and the cells were harvested for experiments.

### Quantitative real-time PCR

#### Genes

Total RNA was extracted using TRIzol® reagent (Gibco, USA) in accordance with the manufacturer’s instruction and then reversed transcribed using the Prime-Script RT Reagent Kit (TaKaRa, Japan). Quantitative PCR was conducted using an ABI 7500 Real-Time PCR system (Life Tech, USA) with SYBR Green Reagents (TaKaRa, Japan). Glyceraldehyde 3-phosphate dehydrogenase (GAPDH) was used to normalize the gene expression levels. All the primers used for real-time PCR were described in supplementary Table 1. Relative gene expression levels were calculated by the Threshold Cycle (CT) method (2−△△CT method).

### C19MC microRNAs

Total microRNAs were extracted using MiPure Cell/Tissue miRNA Kit (RC201, Vazyme). C19MC microRNAs (miR-517c-3p, miR-517-5p, miR-525-3p) and U6 were reverse transcribed into complementary DNA using miRNA 1st Strand cDNA Synthesis Kit (MR101, Vazyme). Then, 2 µl of cDNA corresponding to C19MC microRNAs or U6 were mixed with components of miRNA Universal SYBR qPCR Master Mix. The analysis was performed using a 7500 Real-Time PCR system under the conditions described according to the specification. The expression of C19MC microRNAs and U6 were determined using the comparative CT method relative to the expression in the reference sample. All the primers used were also described in supplementary Table 1.

### Immunostaining

Trophoblast organoids were washed using the cell recovery solution (Corning, USA) and then fixed in 4% paraformaldehyde for 30 min. After permeation and blocking, primary antibodies against human GATA3 (1:100, Abcam, UK), TFA2A (1:100, Abcam, UK) and TFA2C (1:100, Abcam, UK) were added and incubated at 4℃ overnight. On the next day, the organoids were sequentially incubated with fluorescence-labeled rabbit or mouse secondary antibody. Phalloidine and DAPI were treated orderly. Imaging of the stained organoids was carried out by a ZEISS LSM 700 Confocal Laser Scanning Microscope and the ZEN software.

### Single-cell isolation and Library construction

Organoids cultures were washed in cell recovery solution at 4 ℃ for 60 min. In the co-cultured organoids, the dNKs were firstly collect through gently pipetting the culture and centrifuged the media. After washing in PBS and centrifugation, the organoid pellets were disaggregated in stem pro accutase at 37 ℃ for 5 min for three times. Each disaggregated cell suspension was filtered through a 40-µm sieve.

Single-cell suspensions were subjected to dead cell removal using the commercial kit (130-090-101, Miltenyi) to achieve a cell viability ≥ 80% which was determined by AO/PI staining (RE010212, Countstar). Cell suspensions were then loaded onto the 10X Genomics single cell controller for single cell encapsulation and library preparation using the Chromium GEM single cell 3’ reagent kits v3.1 (Dual Index) following standard manufacturer protocol. Single-cell libraries were sequenced on a DNBSEQ-T7 instrument at a PE150 (Paired-end, dual indexing) mode with a minimum sequencing depth of 50,000 reads/cell.

### Single-cell RNA-seq data analysis

#### Pre-processing

Raw sequencing data were analyzed to obtain the cell-level gene expression data by the genomic suite CellRanger version 3.0 developed by 10X Genomics. The human genome reference GRch38 downloaded from the 10X Genomics website was used [45].

### Quality control, cell clustering and annotation

A total of 38,156 single cells from individual organoids and co-cultured organoid were sequenced and pre-processed using the Seurat R package (version 4.0.1) [46]. To ensure high-quality cells were used in downstream analyses, cells containing fewer than 800 detected genes and greater than 20% mitochondrial DNA content were filtered out. Doublet cells were also removed using the DoubletFinder package (version 2.0.3) [47]. The remaining cells were then normalized, scaled and cell cycle scored. Highly variable genes in individual organoids and co-culture organoid was obtained with the “FindVariableFeatures” function using “vst” method for downstream analyses. Following pre-processing, cells from each organoid sample were merged and integrated using Harmony package (version 0.1.1) [48] for batch effect removal.

Cell clustering was performed in Seurat at a given resolution (1.2 in TO merger EVT-TO, 0.7 in dNKs/EVT-TO) using the first 30 principal components. Manual annotation was performed for the clusters by checking expression of conventional cell type markers which were collected from literatures related to the maternal-fetal interface, as well as marker genes obtained via the “FindAllMarkers” function using a model-based analysis of single cell transcriptomics (MAST) GLM-framework implemented in Seurat. The differentially expressed genes (DEGs) in each specific cluster were selected with a minimum log fold change value > 0.2 and a P-value < 0.05. Cell projections were similarly visualized by uniform Manifold Approximaion and Project (UMAP) and t-Distributed Stochastic Neighbor Embedding (t-SNE).

### Mapping and annotating using in vivo placenta atlases reference

To identify transcriptomic similarities with the in vivo placenta reference, the Seurat R package (version 4.0.1) [46] was used to project our scRNA-seq data to public dataset, as described previously [49]. Firstly, “FindTransferAnchors” function was used to identify anchors. The “RunUMAP” function was used to compute the reference UMAP model. Then, the “MapQuery” function was used to project the query data onto the UMAP of the reference which wrapped the following three functions: “TransferData” for cell type label transfer and ADT value imputation; “IntegrateEmbeddings” for reference and query integration by correcting the query’s projected low-dimensional embeddings; “ProjectUMAP” for projection of the query data onto the reference UMAP.

### Gene ontology and Kyoto Encyclopedia of genes and genomes analysis

Gene ontology (GO) [50]and Kyoto Encyclopedia of Genes and Genomes (KEGG) [51] enrichment analysis of significant DEGs for each specific clusters were performed using the ClusterProfiler R package (version 3.18.1) [52]. The enriched terms (Benjamini-Hochberg corrected P-value < 0.05) were visualized using the simplifyEnrichment R package (version 1.8.0) [53].

### Pseudotime trajectory analysis

The Monocle2 R package (version 2.18.0) [54] was used to explore the differentiation of progenitor CTB to specialized STB and EVT, as well as the dNKs differentiation. The integrated raw counts from TO and EVT-TO were loaded into Monocle2 for semi-supervised single cell ordering in pseudo-time, using CDX2 and SPINT1 expression to denote the origin and ERVFRD-1 and HLA-G expression to indicate progress towards STB and EVT, respectively. ITGA2 and NOTCH1 expression were used to identify CCC, named the EVT progenitor. Branched expression analysis modeling (BEAM) was applied to observe the expression of the CTB progenitor gene markers along pseudo-time trajectory towards either STB and or to EVT through CCC. Results were visualized using the “plot_cell_trajectory” function.

### Cell-cell interaction analysis

Cell-cell communication analysis was performed using CellPhoneDB package (version 3.1.0) [55]. Enriched receptor-ligand interactions between two cell types were inferred by the receptor expression in one cell type and the corresponding ligand expression in another cell type. Only receptors and ligands expressed in more than 10% of the cells in the specific cluster were considered for further assessment of cellular crosstalk between different cell types. Pairwise comparisons between all cell types were performed under randomly permuted the labeled clusters 1,000 times and a P-value at 0.05. Biologically relevant receptor-ligands were manually picked among the prioritized interactions according to the number of significant pairs.

### Single-cell regulatory network inference and module analysis

Single-cell regulatory network inference and clustering (SCENIC) package (version 0.12.1) [56] was applied to construct a regulatory network involving trophoblast differentiation-associated transcription factors (TFs) and its downstream targets. Each TF has its regulon activity score (RAS) calculated from each cell. Then, the similarity of the overall regulatory activity of different TFs was assessed and quantified their correlations among cell types by counting the regulon specificity score (RSS). Regulon modules were identified based on the Connection Specificity Index (CSI) to recognize regulons specific associating partners. Network visualization was done using Cytoscape (version 3.9.1).

### Statistical analyses

SPSS Statistics version 25.0 (IBM) was used for the statistical analysis. Data was graphically depicted as the means ± standard deviations (SD) via GraphPad Prism 8.0. Independent t-test and one-way ANOVA analysis of variance were performed to analyze the differences between two groups and multiple groups, respectively. Each experiment was repeated at least three times. P-value < 0.05 was considered as statistically significant.

### Electronic supplementary material

Below is the link to the electronic supplementary material.

**Figure S1**. Cell type-specific regulon activity analysis. A. Same as Figure 5A but for CTB clusters. B. Same as Figure 5A but for STB clusters. C. Same as Figure 5A but for TO and EVT-TO group. **Figure S2**. Activation of regulon modules in different cell types. Related to Figure 5. A. Regulon association network based on CSI matrix. Colors represent each regulon modules. B. Average activity scores of 4 regulon modules in different cell types. **Figure S3**. Decidual natural killer cells isolation and co-culture with EVT-TO. A. Gating strategy for fluorescence-activated dNKs sorting. B. Image of dNKs in individual culture. C. Expression of killer receptors in dNKs. D. Immunofluorescence staining images of GATA3, CD56 and GNLY in dNKs-EVT-TOs co-culture. **Figure S4**. Heatmap showing z-scores of the mean log-trandsormed, normalized expression of genes annotated as selected ligands and receptors expressed in dNKs-EVT-TO co-cultures. A for EVTs. B for dNKs.

**Supplementary Table 1**. Nucleotide base sequences designed in ShangHai ShengGong company.

## Data Availability

All the raw sequencing data of scRNA-seq are accessible on the CNCB-NGDC repository (PRJCA017374).
