# Supplementary material for: Single-cell characterization of self-renewing primary trophoblast organoids as modeling of EVT differentiation and interactions with decidual natural killer cells
Source: BMC Genomics. 2023 Oct 18;24:618. doi: 10.1186/s12864-023-09690-x (PMC10583354; doi:10.1186/s12864-023-09690-x)
Supplement: Supplementary file 1 — Figure S1. Cell type-specific regulon activity analysis. A. Same as Figure 5A but for CTB clusters. B. Same as Figure 5A but for STB clusters. C. Same as Figure 5A but for TO and EVT-TO group. Figure S2. Activation of regulon modules in different cell types. Related to Figure 5. A. Regulon association network based on CSI matrix. Colors represent each regulon modules. B. Average activity scores of 4 regulon modules in different cell types. Figure S3. Decidual natural killer cells isolation and co-culture with EVT-TO. A. Gating strategy for fluorescence-activated dNKs sorting. B. Image of dNKs in individual culture. C. Expression of killer receptors in dNKs. D. Immunofluorescence staining images of GATA3, CD56 and GNLY in dNKs-EVT-TOs co-culture. Figure S4. Heatmap showing z-scores of the mean log-trandsormed, normalized expression of genes annotated as selected ligands and receptors expressed in dNKs-EVT-TO co-cultures. A for EVTs. B for dNKs. [file 12864_2023_9690_MOESM1_ESM.docx]

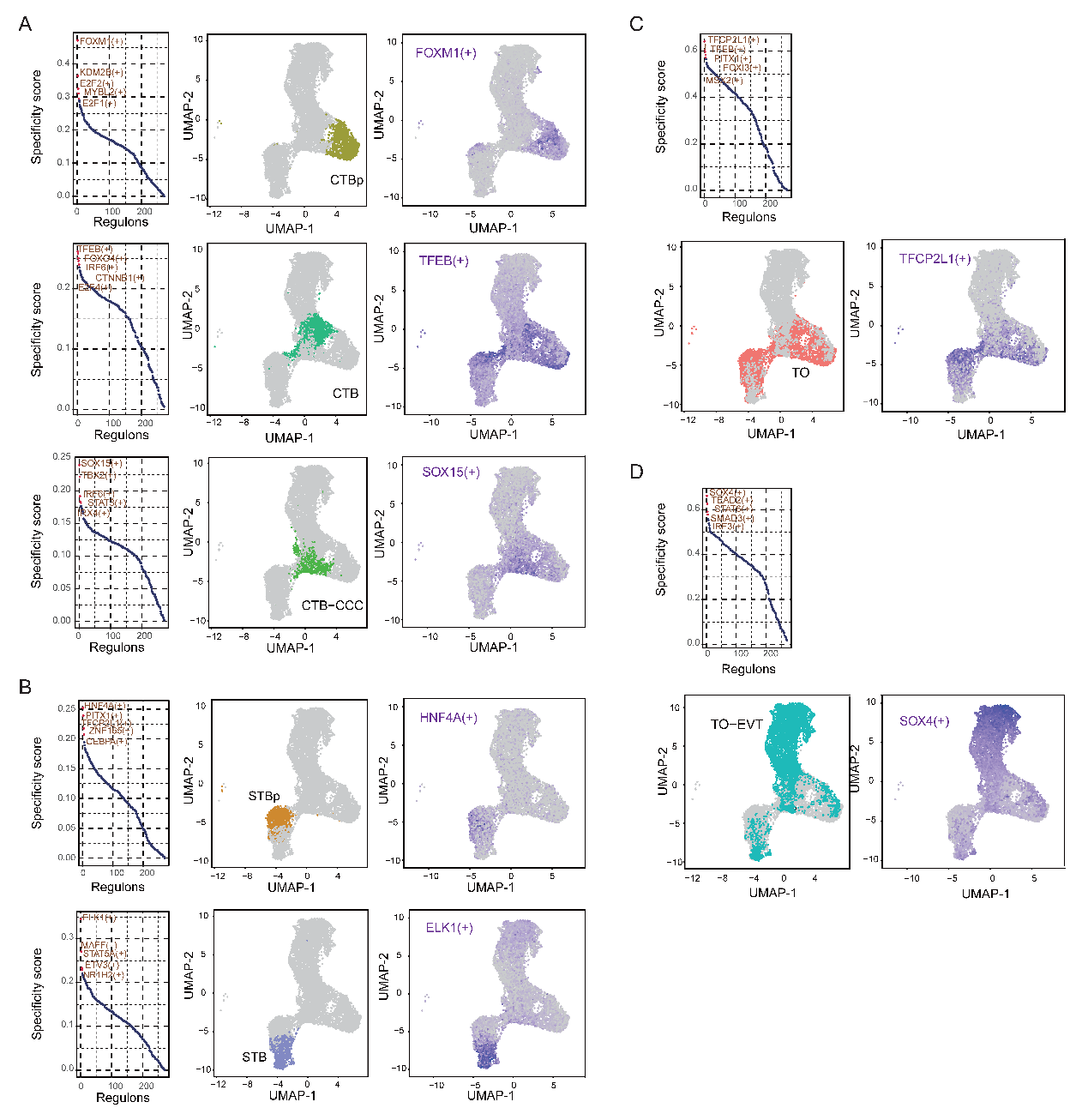


Figure S1. Cell type-specific regulon activity analysis. A. Same as Figure 5A but for CTB clusters. B. Same as Figure 5A but for STB clusters. C. Same as Figure 5A but for TO and EVT-TO group.


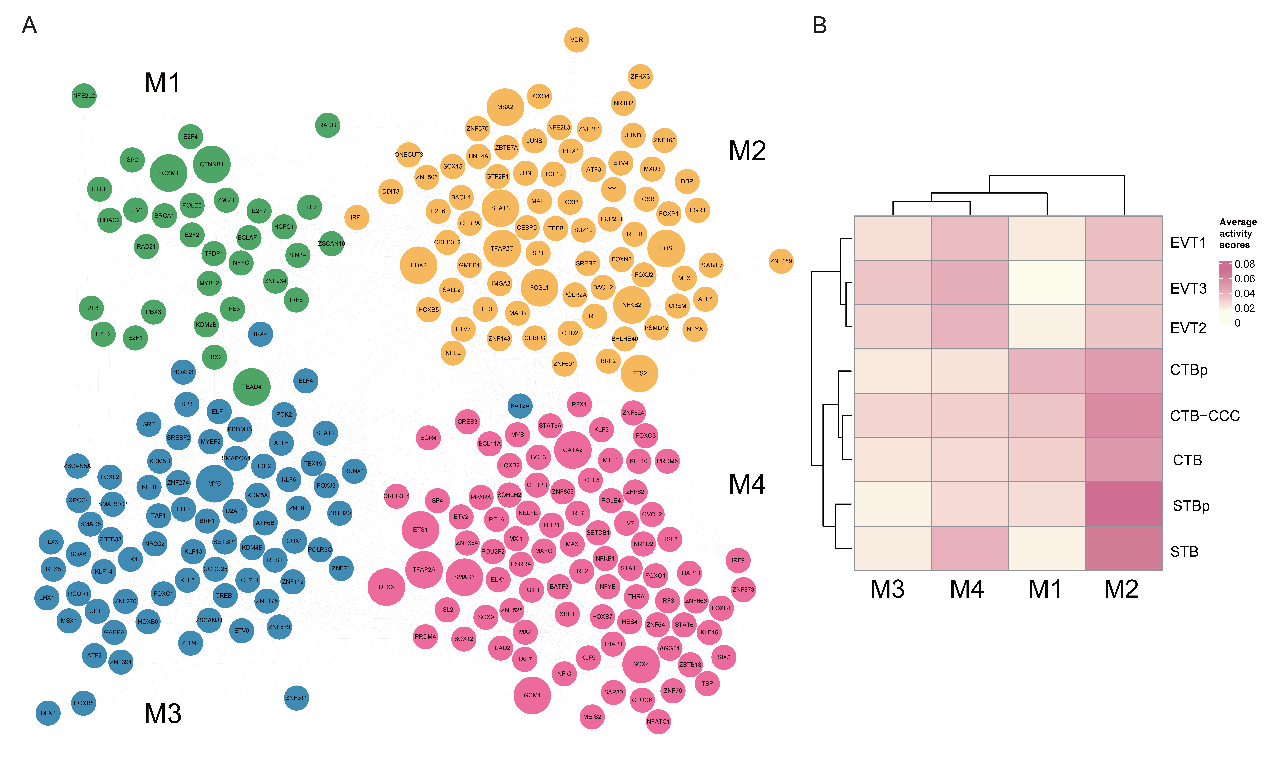


Figure S2. Activation of regulon modules in different cell types. Related to Figure 5. A. Regulon association network based on CSI matrix. Colors represent each regulon modules. B. Average activity scores of 4 regulon modules in different cell types.


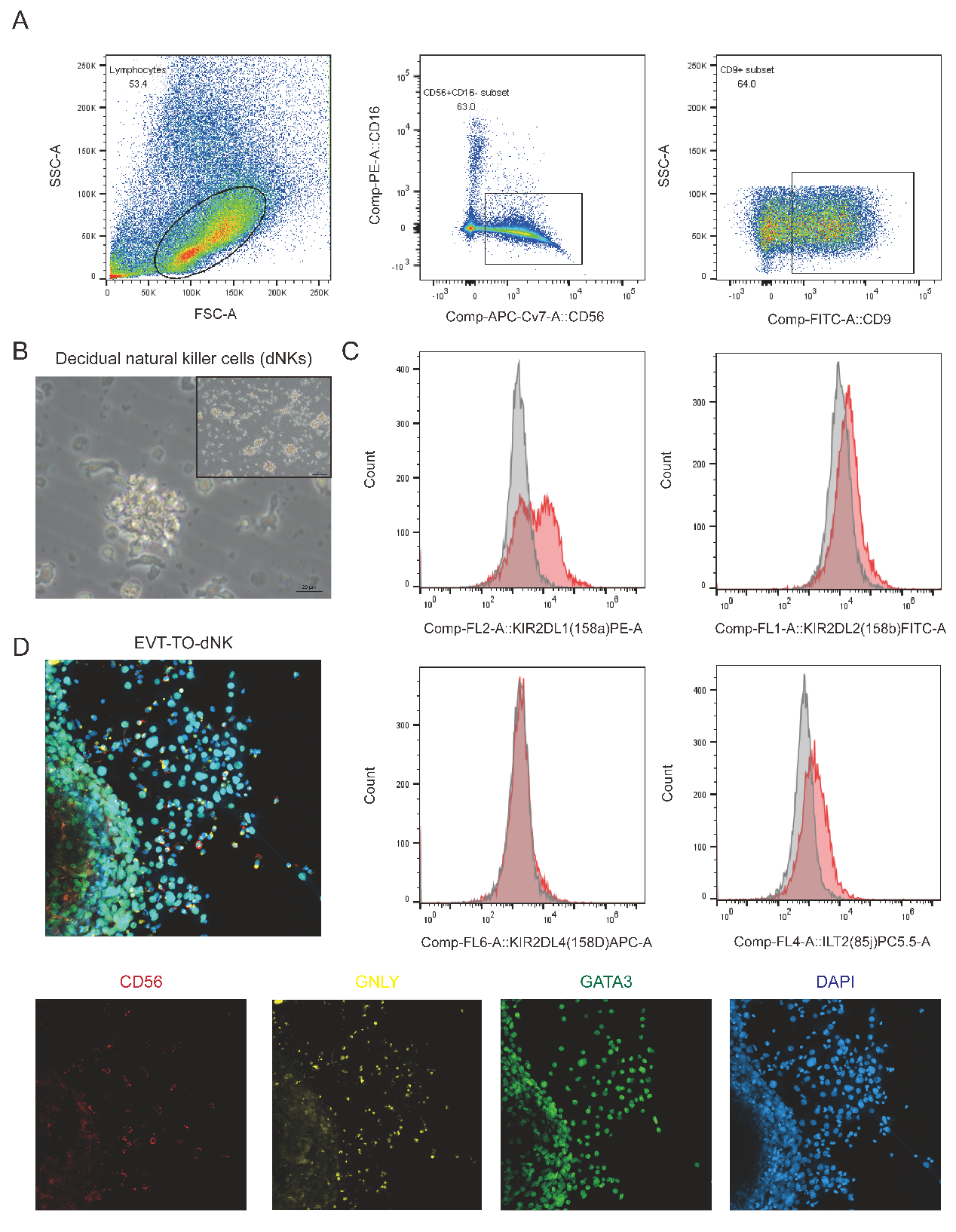


Figure S3. Decidual natural killer cells isolation and co-culture with EVT-TO. A. Gating strategy for fluorescence-activated dNKs sorting. B. Image of dNKs in individual culture. C. Expression of killer receptors in dNKs. D. Immunofluorescence staining images of GATA3, CD56 and GNLY in dNKs-EVT-TOs co-culture.


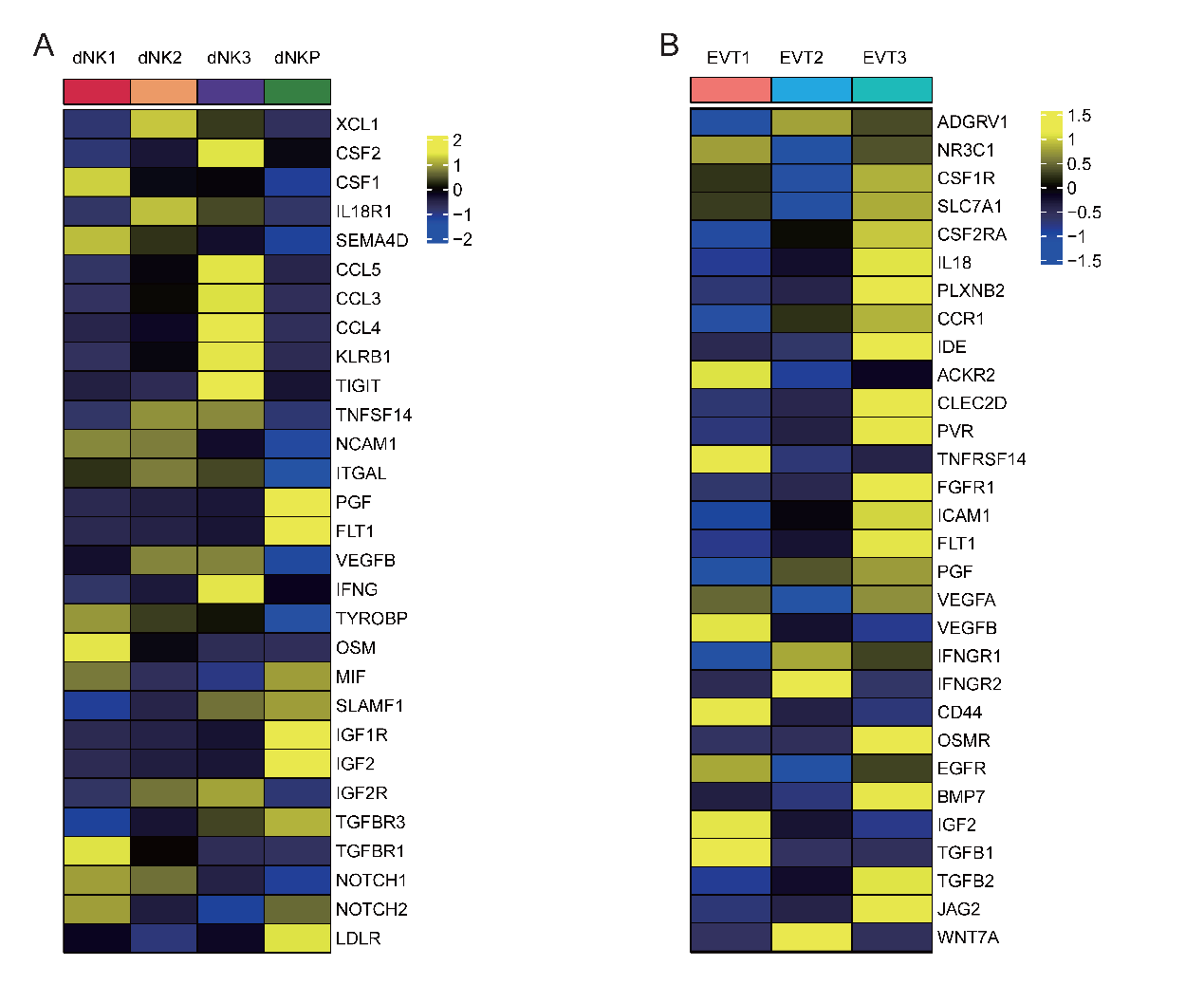


Figure S4. Heatmap showing z-scores of the mean log-trandsormed, normalized expression of genes annotated as selected ligands and receptors expressed in dNKs-EVT-TO co-cultures. A for EVTs. B for dNKs.
