# Supplementary material for: Single-cell characterization of self-renewing primary trophoblast organoids as modeling of EVT differentiation and interactions with decidual natural killer cells
Source: BMC Genomics. 2023 Oct 18;24:618. doi: 10.1186/s12864-023-09690-x (PMC10583354; doi:10.1186/s12864-023-09690-x)
Supplement: Supplementary file 2 — Supplementary Table 1. Nucleotide base sequences designed in ShangHai ShengGong company. [file 12864_2023_9690_MOESM2_ESM.docx]

Supplementary Table1. Nucleotide base sequences designed in ShangHai ShengGong company.

| **1.1 RT-PCR Primers:** | | |
| --- | --- | --- |
| Gene | FORWARD (5’>3) | REVERSE (5’>3) |
| ITGA6 | GAGCATGATGAAAGTCTCGTTC | ACTTCATGTCTCTCTTCAGCAA |
| VGLL1 | CCAAAGGCAAACAGAAGCCTA | CATCACACCTTCACTCTGACTC |
| TP63 | AGGACACGTCGAAACTGTGC | GGACCAGCAGATTCAGAACGG |
| HLA-A | CGAGGATGGCCGTCATGGCG | CACATTCCGTGTCTCCTGGTCCC |
| HLA-B | CAGTTCGTGAGGTTCGACAG | CAGCCGTACATGCTCTGGA |
| HLA-C | GGAGACACAGAAGTACAAGCGC | ACATCCTCTGGAGGGTGTGAGA |
| ITGA5 | CATGATGAGTTTGGCCGATTTG | CCCCCAGGAAATACAAACACTA |
| MMP2 | CAGTACCGAGAGAAAGCCTATT | CAGGATGTCATAGGTCACGTAG |
| PRG2 | TAGTCAAGCTTGGTTTACTTGC | TGACAGAACACTGGATTCGATA |
| **1.2 C19MC microRNAs Primers:** | | |
| *microRNA reversed transcription* | | |
| miR-517c-5p | CTCAACTGGTGTCGTGGAGTCGGCAATTCAGTTGAGAGACAGTG | |
| miR-526-3p | CTCAACTGGTGTCGTGGAGTCGGCAATTCAGTTGAGCGCTCTAA | |
| U6 | CTCAACTGGTGTCGTGGAGTCGGCAATTCAGTTGAGAAAAATATGG | |
| *microRNA qRT-PCR* | | |
| miR-517c-5p | ACACTCCAGCTGGGCCTCTAGATGGAAGCA | |
| miR-526-3p | ACACTCCAGCTGGGGAAAGTGCTTCCTTTT | |
| U6 | ACACTCCAGCTGGGCAAGGATGACACGCAAAT | |
| miR-525-3p | | |
| GTTGGCTCTGGTGCAGGGTCCGAGGTATTCGCACCAGAGCCAACCGCTCT | | |
| GTTGAAGGCGCTTCCCTTT | | |
| Universal reverse primer | | |
| GTGCAGGGTCCGAGGT | | |
